# Dual-target magnetic anomaly detection and recognition based on a board-level micro fully integrated fluxgate tensor for unexploded ordnance (UXO) mission

**DOI:** 10.1038/s41378-026-01227-y

**Published:** 2026-04-07

**Authors:** Zhan Pu, Dongming Fang, Yuhan Dai, Chong Lei

**Affiliations:** 1 National Key Laboratory of Advanced Micro and Nano Manufacture Technology, Shanghai Jiao Tong University, Shanghai, 200240 China; 2 School of Integrated Circuits (School of Information Science and Electronic Engineering), Shanghai Jiao Tong University, Shanghai, 200240 China; 3 Beijing Smartchip Microelectronics Technology Co., Ltd., Beijing, CO 102299 China

**Keywords:** Nanosensors, Sensors

## Abstract

To meet the application requirements for detecting UXO using magnetic detection, the micro fluxgate tensor technology has shown significant value in target recognition, localization, and interference resistance. A board-level micro fluxgate tensor is developed using heterogeneous multi-dimensional integrated triaxial fluxgate technology, achieving synchronous detection and identification of dual targets. The micro fluxgate tensor consists of a typical cross-array formed by four MEMS integrated triaxial fluxgate sensors bonded onto a PCB board, with the size of each triaxial sensor being 17.4 mm × 13.3 mm × 13.8 mm, and the overall size of the micro fluxgate tensor being 86 mm × 80 mm × 16 mm. The micro fluxgate tensor uses a total of 12 uniaxial MEMS fluxgate chips, the size of each chip being 10.8 mm × 6mm × 0.5mm, with an average sensitivity of approximately 1930 V/T and a noise power spectral density below 0.05 nT/√Hz @1Hz. Within a test area of 1.2m × 1.2 m, two differently shaped magnetic targets, an olive-shaped magnet and a spherical magnet, are detected by the micro fluxgate tensor successfully. By comparing the magnetic tensor figure aspect ratios of the olive-shaped magnet (175%) and the spherical magnet (122%), the two targets are distinguished. Furthermore, the magnetic field tensor detection of coexisting cylindrical and spherical magnets is performed, and the results of the magnetic tensor figure indicate the presence of both targets and achieve identification differentiation based on shape aspect ratios, with aspect ratios of 241% and 132% respectively. The micro fluxgate tensor will have advantages such as integration, miniaturization, lightweight design, and low power consumption. It will be more suitable for deployment on portable platforms and unmanned systems, thereby enhancing the efficiency of UXO detection.

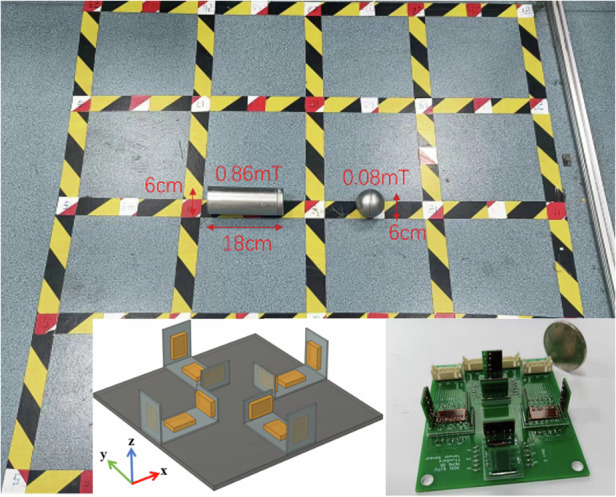

## Introduction

Magnetic Anomaly Detection (MAD) serves as a core technology for locating unexploded ordnance (UXO) by detecting distortions in the geomagnetic field caused by ferromagnetic targets^1,2^. Current magnetic sensing technologies primarily include proton precession magnetometers, superconducting quantum interference devices (SQUID), anisotropic magnetoresistance (AMR) sensors, and fluxgate magnetometers^3–6^. Proton magnetometer is limited by spatial resolution and sampling rate. SQUID requires a low-temperature environment making it difficult to deploy in the field. AMR has a narrow dynamic range and weak noise resistance. In contrast, fluxgate sensors achieve a balance between sensitivity, environmental adaptability, and deployment flexibility, allowing mobile detection at room temperature conditions^7^. Magnetic gradient tensor (MGT) based on the triaxial fluxgate sensor spatial array further enhances detection performance in complex environments^8^. By measuring MGT rather than the total field, it significantly improves the detection advantage of multiple UXOs. The fluxgate tensor can effectively distinguish the superimposed anomalous signals of densely distributed targets by suppressing environmental noise and analyzing the spatial gradient information of the magnetic field, thereby improving the accuracy of target recognition and positioning. This characteristic makes the fluxgate tensor a significant advancement in high-resolution, high-efficiency UXO detection.

Magnetic tensor detection technology has been extensively studied in the field of UXO. In 2001, Nelson proposed a multisensor towed array detection system^9^, which includes magnetometer and electromagnetic induction (EMI) sensor arrays, with a detection probability of 0.95 or higher for UXO. In 2011, Tomasz utilized man portable vector (MPV) and time-domain electromagnetic multi-sensor towed array detection system (TEMTADS) sensor arrays to simultaneously invert the position^10^, orientation, and polarizability of multiple UXOs. In 2013, Li proposed an aeromagnetic survey sensor array^11^, which employed a stable downward continuation method to enhance the resolution of magnetic data, separate overlapping anomalies, and thus significantly increase the number of detected targets. In 2016, Zhou proposed a magnetic anomaly inversion method based on analytical signals^12^, which solved the problem of reduction to the pole in low-latitude regions, with a burial depth inversion error ≤5.5%. In 2019, Sui constructed a cross-shaped five-sensor array with a baseline of 0.1–0.5 m^13^, achieving a positioning error <0.3 m and verifying the advantages of MGT in resisting background interference. In 2022, Sui further developed a vehicle-mounted transient electromagnetic-MGT fusion system^14^, which identifies targets using electromagnetic attenuation characteristics, realizing a positioning error of ≤10 cm for UXOs buried at 2 m depth with a false alarm rate approaching zero. In 2024, Yan adopted a cubic array composed of 8 triaxial magnetometers^15^, achieving stable detection under platform shaking by adaptively integrating search energy.

The system architecture of fluxgate tensor magnetometers has undergone significant evolution. Early large-scale arrays, such as the triangular configuration (side length 40 cm) developed by the Naval Surface Warfare Center in 2002^16^, the tetrahedral setup (side length 1 m) by the U.S. Geological Survey in 2005^17^, the hexahedral array (spacing 0.2 m) by the National University of Defense Technology in 2012^18^, and the cross-shaped gradiometer (baseline 35 cm) by the National Space Science Center in 2019^19^, established the foundation for magnetic field tensor measurements but were constrained by dimensions exceeding 0.5 m, limiting deployment flexibility. In 2022, Han achieved a 96.3% target identification accuracy with a cubic array (400 mm spacing)^20^. However, traditional three-component fluxgate sensors with dimensions approaching 10 cm, combined with the requirement for baseline distances exceeding sensor sizes, have resulted in tensor magnetometer structures often exceeding several tens of centimeters, leading to large volumes, low integration levels, and significant installation errors that hinder engineering applications^21^.

Progress in miniaturization of fluxgate tensor magnetometers has been limited^22–25^. In 2012, Griffin developed a 40 cm^3^ fluxgate gradiometer probe (noise <1 nT/m/√Hz@1 Hz)^26^. In 2015, Janosek integrated 10 sensors into a 46 mm cube^27^, yet manufacturing complexity impeded mass production. Micro-integration triaxial fluxgate technology based on MEMS heterogeneous multi-dimensional integration technology provides a good development direction for the miniaturization of fluxgate tensor. In 2015, Lu proposed a planar triaxial sensor with a Z-axis noise of 6.29 nT/√Hz@1Hz^28^. In 2024, Ma achieved a fully integrated triaxial chip (17.4 × 13.3 × 13.8 mm) using PCB heterogeneous integration^29^, featuring a sensitivity of ≈1949 V/T, noise <0.087 nT/√Hz@1 Hz, and resolving orthogonal packaging challenges through a U-shaped bonding structure. In 2024, Dai introduced a T-shaped micro triaxial sensor (25.7 × 14.8 × 16 mm) with a sensitivity of ≈1070 V/T^30^, noise <0.14 nT/√Hz@1 Hz, and orthogonality deviation <1°. In 2025, Dai further presented a commercial compact L-shaped sensor (12 × 11.7 × 7.2 mm) with a sensitivity of ≈885 V/T^31^, noise <0.085 nT/√Hz@1 Hz, temporal drift <22 nT, and orthogonality error <1°.

Based on MEMS micro triaxial fluxgate sensors, a micro fluxgate tensor array can be realized through integrated manufacturing. In this study, we used four U-shaped triaxial MEMS fluxgate sensors bonded onto a PCB substrate to form a cross-shaped board-level micro fluxgate tensor for experimental research on magnetic tensor detection of targets of different shapes. We achieved synchronous detection and identification of two magnetic targets using the micro fluxgate tensor. Leveraging the miniaturization and integration advantages provided by MEMS technology, the MEMS fluxgate tensor maintains high precision while effectively reducing size, enhancing spatial resolution of magnetic targets, making it suitable for UXO detection tasks.

## Structure and methods

Magnetic tensor is magnetic field gradients of three-component (Bx, By, Bz) of magnetic field B in three directions, which constitute a tensor matrix G with nine components, as it can be seen in formula 2-1.

1 $$G=\left[\begin{array}{cc}{Bxx} & \begin{array}{cc}{Byx} & {Bzx}\end{array}\\ \begin{array}{c}{Bxy}\\ {Bxz}\end{array} & \begin{array}{cc}\begin{array}{c}{Byy}\\ {Byz}\end{array} & \begin{array}{c}{Bxy}\\ {Bzz}\end{array}\end{array}\end{array}\right]$$

Since the curl and divergence of the magnetic field are both zero and its tensor matrix is symmetric, which is shown in formula 2-2 and 2-3, only five independent components (Bxx, Byx, Byy, Bzx, Bzy) need to be measured to get the complete tensor matrix.

2 $$\nabla \times B=0$$

3 $$\nabla \cdot B=0$$

Numerous existing results have demonstrated that the cross-shaped architecture of tensor arrays has the minimum theoretical measurement error compared to other architectures such as tetrahedral, triangular, and hexahedral^8^. Combining our previous research on MEMS uniaxial fluxgate chips and triaxial integrated fluxgate chips^31,32^, we will first manufacture the MEMS uniaxial fluxgate chip, then integrate the manufacturing of the triaxial fluxgate chip, and finally bond four triaxial fluxgate chips symmetrically in a cross-shaped architecture on a PCB substrate to form an integrated miniaturized fluxgate tensor.

**Fig. 1:**
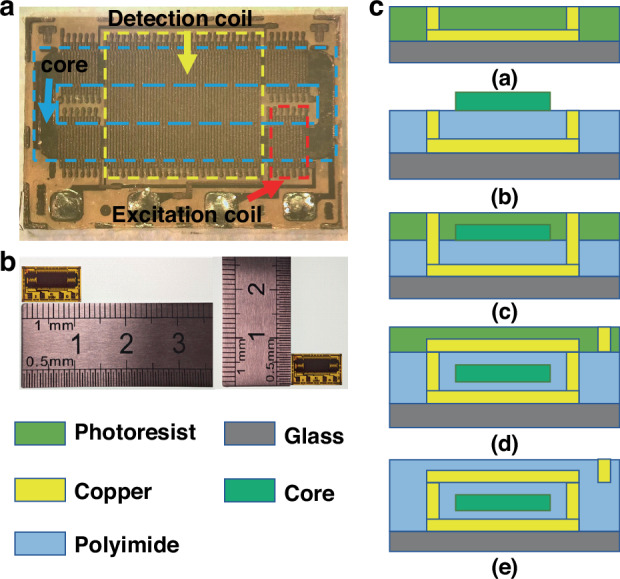
Design and manufacturing of MEMS uniaxial fluxgate sensor chip. **a** Structure, **b** dimension, **c** simplified manufacturing process

Based on previous MEMS fluxgate technology accumulation, this study designed a uniaxial fluxgate sensor suitable for high-precision geomagnetic detection. As shown in Fig. 1a, the sensor employs a racetrack-shaped amorphous soft magnetic thin strip core and parallel fluxgate working principle, integrating four differential excitation coils and one sensing coil, forming a three-dimensional solenoid structure. As shown in Fig. 1b, the design dimensions of the sensor are 10.8 mm × 6 mm. Its fabrication process combines thick photoresist lithography and multi-layer electroplating technology, as shown in Fig. 1c. During manufacturing, the AZ P4620 photoresist is first exposed to UV light using a SUSS MA6 photolithography machine to form a 20 μm thick mold. Copper bottom coil is electroplated at 120 mA current using a sulfate copper plating process. The photolithography and electroplating steps are repeated to prepare 50 μm × 100 μm rectangular cross-section vias. A 5 μm thick polyimide is spin-coated as an insulating layer, which is then cured under vacuum environment ( < 10⁻³ Pa) at 270 °C for 2 h, reducing residual stress below 30 MPa. Using a micro-manipulation robot, cobalt-based amorphous thin strip racetrack-shaped magnetic cores, are sequentially affixed to designated positions to form a closed magnetic circuit. Copper vias are electroplated again to exceed the thickness of the magnetic core, ensuring complete encapsulation of the core. Polyimide is reapplied and spun to protect the core and vias before electroplating the top copper coil. Finally, polyimide is reapplied and cured once more, followed by chemical mechanical polishing (CMP) to precisely control surface flatness and expose pad areas. This process achieves interlayer alignment accuracy of ±1 μm on a 4-inch wafer, while optimizing electroplating parameters keeps coil resistance deviation within ±8%, significantly improving the consistency of the sensor chip.

The actual fabricated MEMS uniaxial fluxgate chips are 10.8 mm × 6 mm × 0.5 mm after the glass substrate has been thinned. The excitation coil comprises 60 turns with a resistance of 2 Ω, while the detection coil consists of 59 turns and has a resistance of 3 Ω. The core dimensions are 600 μm in width and 15 μm in thickness.

**Fig. 2:**
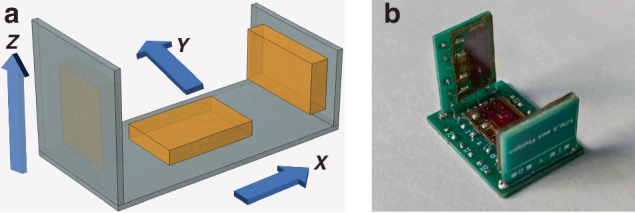
U-shaped integrated triaxial fluxgate sensor. **a** Model, **b** physical photo

Based on heterogeneous multi-dimensional integration technology, we bond the fluxgate chip to the corresponding substrate to form a component module. The alignment accuracy is ensured by aligning the T-shaped marks on the chip and the PCB substrate. The three component modules are orthogonally connected through column hole structures, with Y and Z component substrates mounted on the X component substrate to form a U-shaped structure, as shown in Fig. 2a. After welding fixation inside the holes, an integrated micro fluxgate sensor capable of measuring the three components of the geomagnetic field vector is obtained. The manufactured triaxial sensor has dimensions of 17.4 mm × 13.3 mm × 13.8 mm, with a machining error less than 50 μm, according to the standard error for mechanical cutting of PCB substrates, as shown in Fig. 2b. This design offers significant advantages in size and weight compared to traditional fluxgate sensors.

**Fig. 3:**
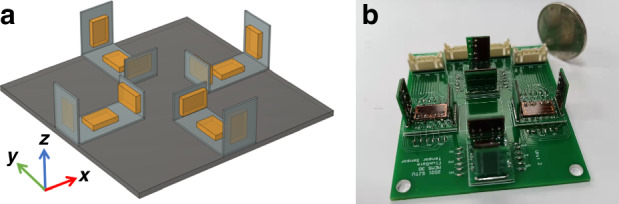
The MEMS fluxgate tensor. **a** Model, **b** physical photo

Using the obtained micro triaxial sensor as an array unit, we further designed a classic cross-shaped PCB-level micro fluxgate tensor, as shown in Fig. 3a. Four U-shaped triaxial sensors are used, aligned using border patterns and bonded fixedly at the center in a full symmetrical manner on the PCB substrate. The baseline distance between sensors is 20 mm. Ultimately, a MEMS micro fluxgate tensor with dimensions of 86 mm × 80 mm × 16 mm is obtained, as shown in Fig. 3b. The miniaturization advantage of the sensor units also effectively shortens the baseline distance design requirement for the tensor array, thus achieving the overall miniaturization of the fluxgate tensor, enhancing its application adaptability, and expanding its application range.

## Experimental samples

### Calibration of basic performances for MEMS uniaxial fluxgate chip

**Fig. 4:**
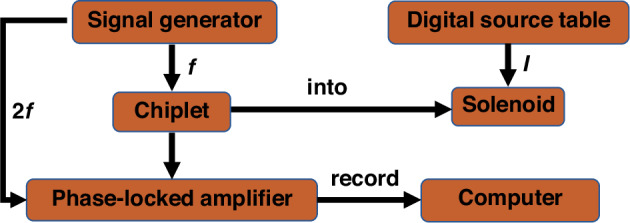
Schematic diagram of fluxgate sensor open loop testing system

The fundamental performance of twelve MEMS uniaxial fluxgate chips used in tensor arrays is tested and calibrated according to the classical second-harmonic method. As illustrated in Fig. 4, we set up an open-loop testing system to test MEMS fluxgate chips. A function generator (Tektronix AFG 3022) serves as the sinusoidal excitation signal source, while a power amplifier (NF BA4825) supplies adequate excitation current. For measuring the second harmonic output voltage of the chips, a lock-in amplifier (Stanford SR844) is utilized. A solenoid positioned within a six-layer shield connected to a source meter (KEITHLEY 2450) provides an accurate test magnetic field. Additionally, a dynamic signal analyzer (Keysight 35670 A) is used to analyze the sensor noise.

Systematic experiments were conducted within the frequency optimization range of 100 kHz–1 MHz and the current testing range of 30 mA–100 mA. The results show that the sensor achieves the optimal performance when the excitation frequency is 400 kHz and the excitation current is 60 mA. In these optimal operating conditions all chips are tested, including sensitivity, range, and noise power spectrum density, as shown in Table 1. The average sensitivity of the twelve chips is about 1930V/T with an error of less than 5%, and noise power spectrum density of all chips is lower than 0.05nT/√Hz@1 Hz. The range of chips is all 60 μT. Given that the power consumption of a single chip is 8 mW, the power consumption of the entire fluxgate tensor array is 96 mW.

**Table 1:** Performances of the fluxgate chips

	Sensitivity (V/T)			Noise (nT/√Hz@1 Hz)		
Tensor Unit	X	Y	Z	X	Y	Z
U1	1851	1863	1898	0.031	0.042	0.044
U2	1989	1865	1985	0.038	0.036	0.043
U3	1992	1998	2014	0.047	0.037	0.045
U4	2042	1850	1836	0.033	0.041	0.046

### Ferromagnet target detection based on the fluxgate tensor

**Fig. 5:**
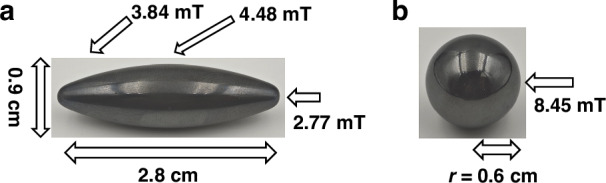
Ferromagnet target. **a** Olive type, **b** spherical type

To validate the effectiveness of the MEMS fluxgate tensor, a square experimental area measuring 1.2 m × 1.2 m is established on the ground. Two different shaped ferromagnets are selected as the target for measurement, as shown in Fig. 5. The first one is an olive type ferromagnet, which serves as the experimental group, mainly made of neodymium-iron-boron material, with dimensions of 28 mm in length and 9 mm in height. The surface magnetic field generated by the olive magnet is measured using a Gaussmeter, yielding values of 4.48 mT at the center and 2.77 mT at both ends. The second one is a spherical ferromagnet, which serves as the control group, also made of neodymium-iron-boron material, with a surface magnetic field of 8.45 mT at the magnetic pole.

Due to the existence of complex magnetic interference sources such as electrical equipment in the laboratory, a location far away from interference sources, with open space, uniform and stable magnetic field distribution, and flat terrain is selected in the laboratory. A 1.2 m × 1.2 m square area is planned using colored tape, and the 25 measurement points are evenly spaced out with 30 cm. In order to ensure the accuracy of the experimental results, four additional measurement points are added between each row of measurement points in the experimental group, and the measurement area is a 5 × 9 distribution square. The tested ferromagnets are positioned at the center of grid area. The N-S orientation of the tested ferromagnets is aligned along its short axis at the center. At a height of 10 cm above the target, the MEMS fluxgate tensor sequentially detected magnetic fields at each grid point. The collected magnetic field signals are recorded by a data acquisition card and subsequently transmitted to a computer for analysis and processing.

**Fig. 6:**
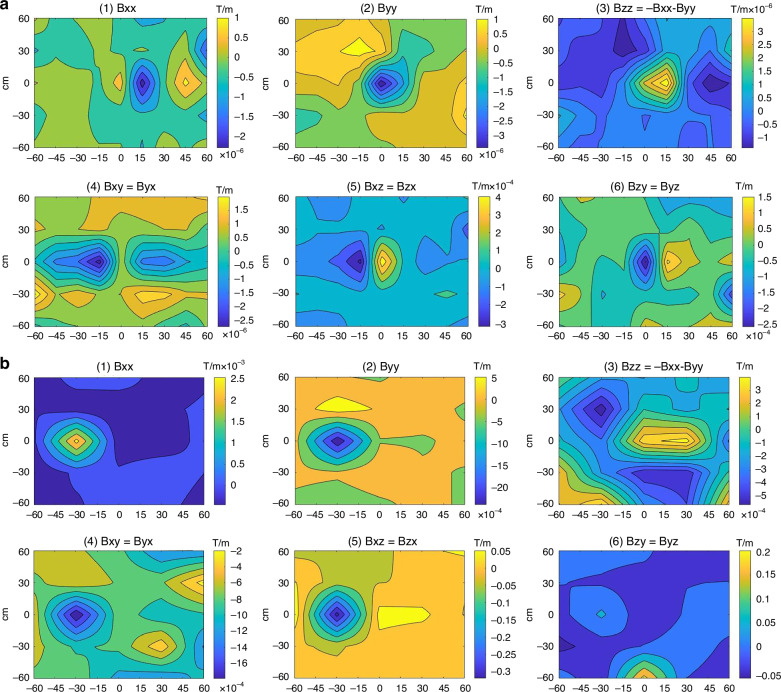
Experimental results of tensor detection. **a** olive type, **b** spherical type

To study the fluxgate tensor detection identification capabilities of ferromagnets with different shapes, magnetic detection is performed on olive type ferromagnet and spherical type ferromagnet. In the magnetic tensor detection experiment, we used experimental parameters with a sampling density of 45 points (5 × 9) to enhance detection accuracy. The data collected is processed to produce six magnetic anomaly tensor cloud maps, which also show the specific magnetic anomaly data and corresponding magnetic tensor distribution within a 1.2 m × 1.2 m area, as shown in Fig. 6.

Compared to the olive ferromagnet target magnetic imaging experimental results in Fig. 6a, the spherical ferromagnet target magnetic imaging experimental results in Fig. 6b exhibit better symmetry. Due to the stronger magnetism of the spherical ferromagnet, the magnetic field intensity values in its magnetic field image are generally higher. As shown in Fig. 6b, the isoclines in the magnetic anomaly tensor figure (Bxx, Byy, Bxy, and Bxz) clearly present a spheroidal shape, closely resembling the theoretical magnetic gradient map of a spherical ferromagnet. The other two figures (Bzz, Bzy) deviate significantly from the theoretical values, due to measurement errors in the Z direction. The magnetic imaging localization results for the spherical ferromagnet and the olive ferromagnet show little difference. We defined the center positioning distance error as the distance between the center point of the target image and the true center point of the target. The shape aspect ratio is defined as the aspect ratio of the magnetic anomaly contour map formed by measuring the target magnetic field gradient through the micro-fluxgate tensor. The center positioning distance error for the olive ferromagnet is 15.42 cm, with a shape aspect ratio of the target image at 175%. In contrast, the spherical ferromagnet has smaller center positioning error and shape aspect ratio, with a center positioning distance error of 14.95 cm, which is 3% lower than that of the olive ferromagnet, and an aspect ratio of 122%, approaching a circular proportion. The experimental results indicate that the micro magnetic tensor meter has a certain ability to identify the boundary shapes of magnets.

### Dual-objective tensor detection

To investigate the ability of the micro fluxgate tensor to simultaneously image multiple magnetic targets, the magnetic targets are replaced with two similarly sized hollow magnets, one cylindrical and one spherical. The cylindrical hollow magnet has a height of 17.5 cm and a diameter of 6 cm, with the magnetic field strength at the center surface poles of approximately 0.08 mT. The spherical hollow magnet has a diameter of 7 cm, with the magnetic field strength at the surface poles of approximately 0.86 mT. Both magnetic targets have relatively small magnetic field strengths.

**Fig. 7:**
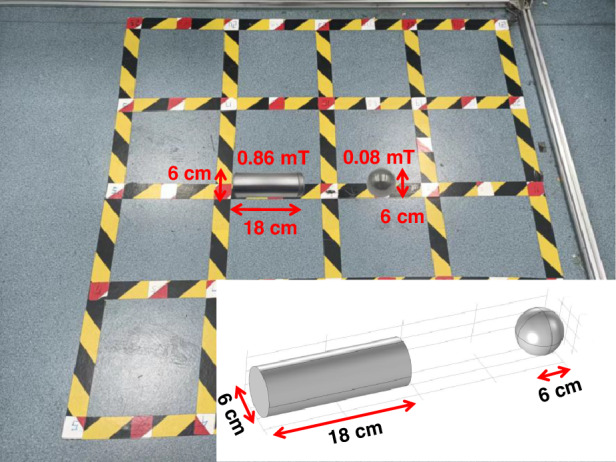
Dual-object magnetic imaging experiment scene layout

As shown in Fig. 7, the test site uses a square grid layout of the same size, with a sampling density of 45 points. The two magnetic targets are placed horizontally at the center of the measurement area, symmetrically about the vertical axis of the site, with the centers of the cylindrical and spherical hollow magnets located at (−30 cm, 0) and (30 cm, 0), respectively.

**Fig. 8:**
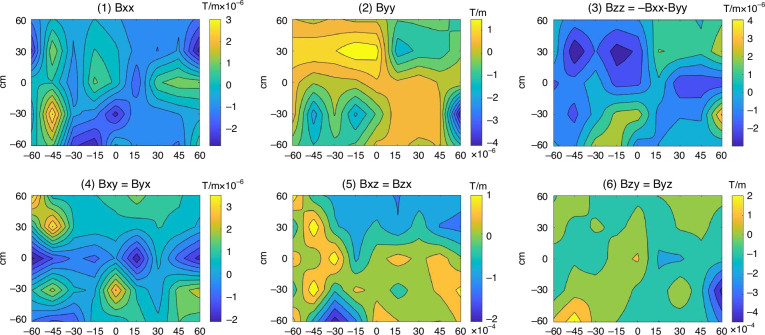
Experimental test results of the dual-object magnetic imaging experiment

Figure 8 shows the magnetic tensor image results of simultaneously detecting two magnetic targets using a micro fluxgate tensor. There are two left-right distributed magnetic anomaly extremums in Byy and Bzz. Bxy can relatively accurately describe the magnetic anomaly information of two differently shaped magnetic targets. For the cylindrical hollow body, its magnetic anomaly contour lines are sparse and rectangular in shape. Specifically, the central positioning distance error is 17.28 cm, the vertical size error and horizontal size error are 60% and 50%, respectively, and the central magnetic tensor value is approximately 2 μT/m. For the spherical hollow body, its magnetic anomaly contour lines are denser and circular in shape. In detail, the central positioning distance error is 14.57 cm, the vertical size error and horizontal size error are 38% and 22%, respectively, and the central magnetic tensor value is approximately 2 μT/m. The center distance error between the two hollow targets is about 60%. The aspect ratios of the cylindrical hollow body and the spherical hollow body are 241% and 132%, respectively.

The experimental results clearly distinguish the presence of cylindrical hollow body and spherical hollow body, and can also identify the differences between the two targets from the shape aspect ratio results. Compared to the detection results of small magnets in the previous section, the aspect ratio detection data for large magnets are closer to the true target data. The results indicate that, in actual testing, the higher the detection density relative to the target size, the more accurate the detection results.

## Conclusion

In conclusion, this paper reports the simultaneous detection and identification of two magnetic targets based on magnetic tensor detection for UXO missions, using a board-level micro fully integrated fluxgate tensor. Based on heterogeneous multi-dimensional integration technology, we design an integrated fluxgate tensor by integrating three MEMS fluxgate chips to form a basic triaxial fluxgate sensor, and four triaxial fluxgate sensors bonded on a PCB substrate to form a fluxgate tensor array. The fabricated MEMS fluxgate tensor is the classic cross-shaped tensor architecture consisting of 4 triaxial fluxgate sensors, that the size of the tensor is 86 mm × 80 mm × 16 mm with each triaxial sensor size of 17.4 mm × 13.3 mm × 13.8 mm and the baseline distance of 20 mm. Performance tests show that the 12 uniaxial MEMS fluxgate chips used in the tensor have an average sensitivity of 1930 V/T, noise power spectrum density <0.05 nT/√Hz@1 Hz, a range of 60 μT, and a total power consumption of 96 mW for the entire tensor.

Magnetic field tensor detection is performed on individual magnetic targets shaped like olive-shaped and spherical, respectively, using the MEMS fluxgate tensor. The results indicate that there are distinct magnetic field tensor characteristics for different magnetic targets for localization and identification, with shape aspect ratios of 175% and 122% respectively, and central distance positioning errors of 15.42 cm and 14.95 cm. Furthermore, for coexisting cylindrical and spherical magnetic targets, the magnetic tensor detection results clearly show the presence of both targets and achieve identification differentiation based on shape aspect ratios, with shape aspect ratios of 241% and 132% respectively, and central distance positioning errors of 17.28 cm and 14.57 cm.

Based on our MEMS fluxgate chip operating at a frequency of 400 kHz, theoretically, a sampling rate of 100 kHz (complying with the Nyquist criterion) can be adopted in the future, effectively enhancing data acquisition speed and density. Subsequently, we will further develop automated high-speed multi-channel data acquisition systems to achieve multidimensional high-resolution synchronous acquisition of magnetic field signals, effectively reducing noise and enhancing the ability to identify weak magnetic anomalies, thereby significantly improving the precision, multi-target resolution capability, and anti-interference performance of UXO detection in complex environments.
